# Research Into Evidence-Based Psychological Interventions Needs a Stronger Focus on Replicability

**DOI:** 10.32872/cpe.9997

**Published:** 2023-09-29

**Authors:** Helen Niemeyer, Christine Knaevelsrud, Robbie C. M. van Aert, Thomas Ehring

**Affiliations:** 1 Department of Clinical Psychological Intervention, Freie Universität Berlin, Berlin, Germany; 2 Department of Methodology and Statistics, Tilburg University, Tilburg, the Netherlands; 3 Department of Psychology, LMU Munich, Munich, Germany; Philipps-University of Marburg, Marburg, Germany

**Keywords:** replicability, evidence-based interventions, criteria development

## Abstract

**Background:**

It is a precondition for evidence-based practice that research is replicable in a wide variety of clinical settings. Current standards for identifying evidence-based psychological interventions and making recommendations for clinical practice in clinical guidelines include criteria that are relevant for replicability, but a better understanding as well refined definitions of replicability are needed enabling empirical research on this topic. Recent advances on this issue were made in the wider field of psychology and in other disciplines, which offers the opportunity to define and potentially increase replicability also in research on psychological interventions.

**Method:**

This article proposes a research strategy for assessing, understanding, and improving replicability in research on psychological interventions.

**Results/Conclusion:**

First, we establish a replication taxonomy ranging from direct to conceptual replication adapted to the field of research on clinical interventions, propose study characteristics that increase the trustworthiness of results, and define statistical criteria for successful replication with respect to the quantitative outcomes of the original and replication studies. Second, we propose how to establish such standards for future research, i.e., in order to design future replication studies for psychological interventions as well as to apply them when investigating which factors are causing the (non-)replicability of findings in the current literature.

## Highlights

- Refined replicability criteria used to identify empirically supported treatments are proposed.
- Concrete steps for refining replication in research on psychological interventions are proposed.
- A taxonomy of direct to conceptual replication adapted to research on interventions is provided.

Recent years have seen an increased focus on conceptual approaches to the replicability of research findings, and a growing number of empirical investigations on this issue, in the areas of psychology (Klein et al., 2014; Klein et al., 2018; Open Science Collaboration [OSC], 2015), economics (e.g., Camerer et al., 2016), epidemiology (e.g., Kaltiala-Heino, Työläjärvi, & Lindberg, 2019; Zisook et al., 2007) and medicine (Errington, Denis, Perfito, Iorns, & Nosek, 2021). Replicability refers to “the ability of a researcher to duplicate the results of a prior study if the same procedures are followed but new data are collected” (Bollen, Cacioppo, Kaplan, Kronsnick, & Olds, 2015; p. 3). Research related to psychological interventions has not paid the same level of attention to recent conceptual developments of replicability (Tackett et al., 2017) as seen in other fields. Yet the strong emphasis on providing evidence-based treatments in clinical psychology and psychiatry (e.g., Tolin et al., 2015) demands that clinical practice should be directly informed and guided by the best available empirical evidence on the efficacy of interventions, as typically collected in randomized controlled trials (RCTs). A precondition for evidence-based practice is that the research is replicable in a wide variety of clinical settings in order to demonstrate high external validity.  Low replicability in a research field may be partly due to so-called “hidden moderators” (Van Bavel, Mende-Siedlecki, Brady, & Reinero, 2016), which prevent the effect from being observed in a replication due to an (unobserved) moderator. Examples include characteristics of the clinical population to which the intervention is offered, treatment-related moderators, or differences in contextual variables. In other words, a study might be successfully replicated in a research outpatient clinic but not in a regular community clinic. Identifying hidden moderators is crucial in order to critically evaluate the generalizability of treatment effects to different clinical settings. “Direct” and “conceptual” are labels for replication studies depending on the similarity to the original study (LeBel et al., 2018; Zwaan, Etz, Lucas, & Donnellan, 2018). Direct replication studies allow to investigate the replicability of a study result, whereas conceptual replications serve to determine the generalizability. The relevance of replication categories has been shown in other fields, such as economics (Fiala, Neubauer, & Peters, 2022; Peters, Langbein, & Roberts, 2018), where different replication rates were found depending on the definition of the replication studies. In order to define the similarity between original and replication study consensus on the most important characteristics is necessary. The ”constraints on generality” criteria (COG; Simons, Shoda, & Lindsay, 2017) help to explicitly determine the targeted population and the study procedures in order to define a direct replication as well as to identify hidden moderators in conceptual studies. A COG statement overcomes the ambiguity of classifying replications as direct or conceptual post hoc because it specifies the target populations for the original claim (Simons et al., 2017; Simons, Shoda, & Lindsay, 2018).

In addition, non-replicability of effects may also be caused by questionable research practices (QRPs; John, Loewenstein, & Prelec, 2012). QRPs comprise a range of activities that are not a research field´s best practices, such as flexibly analyzing data until the results are significant (called *p*-hacking; Whitt et al., 2022) or hypothesizing after the results are known (called HARKing; John et al., 2012). They cause an overrepresentation of statistically significant results in the literature. Performing multiple analyses in combination with selectively reporting statistically significant results increases the number of false-positive findings in the published literature (Forstmeier, Wagenmakers, & Parker, 2017; Simmons, Nelson, & Simonsohn, 2011) and biases effect size estimation. Other factors that may cause non-replicability are reporting errors or sampling error. Importantly, in a given case of non-replicability, more than one factor can be expected to be relevant (Nosek et al., 2022).

Closely related to replicability is reproducibility. Reproducibility is obtained when the reanalysis of the original data using the same procedures arrives at the same result (Maassen et al., 2020). This is also referred to as computational or analytic reproducibility (LeBel et al., 2018). Reproducibility in psychology was investigated by Artner and colleagues (2021) who found that 70% of the reported statistical results were reproducible. When comparing reproducibility rates across disciplines, it is important to note that the definitions of replicability and reproducibility differ across disciplines (Artner et al., 2021). To date, reproducibility attempts are highly uncommon in research on psychological interventions (see also, Sandve, Nekrutenko, Taylor, & Hovig, 2013).

## Do Current Research Standards Pay Enough Attention to Replicability?

Current standards for investigating psychological interventions, identifying evidence-based interventions, and making recommendations for clinical practice in clinical guidelines include criteria that are relevant for the issue of replicability. For example, the criteria for empirically supported treatments (ESTs; David, Lynn, & Montgomery, 2018) were laid down by the American Psychological Association’s (APA) Division 12 in the early 1990s (Chambless & Hollon, 1998; for a recent revision, see Tolin et al., 2015). According to these criteria, treatment effects must have been demonstrated in several independent studies, and a systematic evaluation of the methodological quality of studies as well as risk of bias needs to have been conducted, e.g., using the Cochrane risk-of-bias tool (ROB; Sterne et al., 2019) or the Grading of Recommendations, Assessment, Development and Evaluations (GRADE; Guyatt et al., 2008), consisting of six domains (e.g., risk of bias, [im-]precision of effect estimates). The need to critically assess study quality and the risk of bias has also led to the development of specific reporting standards for clinical trials, such as the Consolidated Standards of Reporting Trials (CONSORT; Schulz et al., 2010), and for reporting systematic reviews and meta-analyses, such as the Preferred Reporting Items for Systematic Reviews and Meta-Analyses” statement (PRISMA; Moher et al., 2015) or the “Meta-Analysis Reporting Standards” (MARS; American Psychological Association, 2020).

However, despite these important advances, the criteria used to identify ESTs and/or recommend clinical interventions for clinical guidelines currently have not yet been updated in line with the recent advances on replicability in the wider field of psychology and in other disciplines (Errington et al., 2021). Although the reporting standards and rating schemes address some of the variables that are relevant to assess (the lack of) replicability in studies on psychological interventions (i.e., pre-specification of the hypotheses and statistical methods, examining publication bias and heterogeneity), they neither include all of the relevant aspects nor do they make an explicit distinction between different types of replication (e.g., direct versus conceptual replications) or specify statistical criteria for a successful replication. A refinement of the criteria for replication in research on psychological interventions and specific suggestions for their application are therefore required. Moreover, an assessment of QRPs, reporting error and demands for pre-registration are currently not included in the quality assessment of clinical studies.

Currently there are only few investigations of the replicability of studies on psychological interventions. One exception is Sakaluk et al. (2019) who systematically examined the evidential value of treatments that have been classified as ESTs by standard criteria. They also applied Schimmack’s replicability index (R-index, Schimmack, 2016), which focuses on statistical significance, and statistical power, as well as Bayesian meta-analysis. Results showed that statistical power and replicability estimates were low. Moreover, differences in the level of empirical support according to EST criteria did not parallel differences in indices of statistical power or replicability. Based on their analysis, the authors argued that higher methodological standards are necessary in research on psychological interventions, including sufficient statistical power and standards for reporting descriptive and inferential statistics.

In line with Sakaluk and colleagues (2019) as well as with the recommendations developed in other areas of psychology and beyond (Ioannidis, 2008; Valentine, 2009), we suggest that there is a need to enhance the replicability of research into psychological interventions and therefore propose to refine the definition of and criteria for replicability in this field. To this aim, some of the developments and resources from other areas will be adopted and, if necessary, adapted to the specificities of research on psychological interventions, as well as the given criteria and definitions refined.

## Proposing a Research Strategy for Assessing, Understanding, and Improving Replicability in Research Evaluating Psychological Interventions

**Figure 1:**
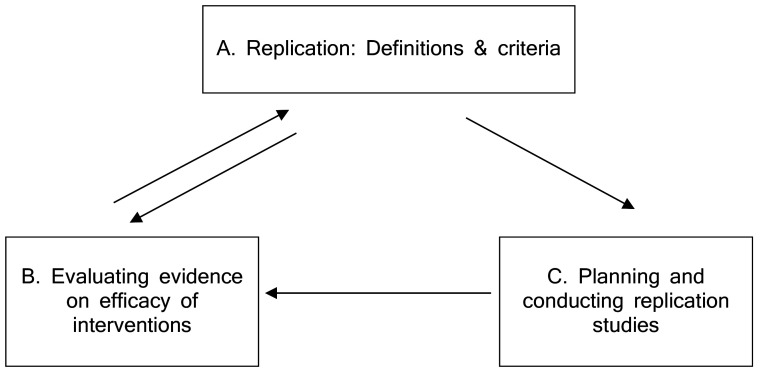
A Strategy for Assessing, Understanding, and Improving Replicability in Research on Psychological Interventions

To improve the current situation, we propose progress in three interrelated areas (A – C; see Figure 1). The concrete steps that need to be taken are described in the following sections.

### A. Replication: Definitions and Criteria

First, the definition of replication currently used in research on psychological interventions is refined, based on a taxonomy of different study design types of replication, study characteristics that increase the trustworthiness of results, and statistical criteria for (un-)successful replication. At a minimum, we suggest three aspects to be crucial:

#### 1. Taxonomy of Replication

Refining replication in research on psychological interventions is a complex endeavor. The definition of replication as aiming to duplicate the results of an original study by applying the same procedures to a new sample (Bollen et al., 2015) provides no specific criteria as to what constitutes "the same procedure” with respect to the characteristics of an original study. Similarly, the EST criteria that treatment effects need to be demonstrated in several independent studies do not specify any details of the study designs of the required independent studies (Tolin et al., 2015).

Attempts to refine the concept of replication have been made in other areas of psychology and social sciences. We adopt the approach of LeBel et al. (2018) who provide a replication taxonomy ranging from direct to conceptual replication, depending on the degree of similarity between an original and a replication study according to several design facets, such as the operationalization of the independent and dependent variables, or investigator independence. To investigate the replicability of a treatment effect, direct replications are necessary. Conceptual replications cannot falsify the hypothesis of replicability, but can, on the other hand, help to evaluate the boundary conditions of treatment effects, the generalizability of intervention effects to different contexts, and/or the mechanisms of change underlying treatment effects. They can help to answer the question of whether (and which) hidden moderators are a cause of low replicability in “combination” with direct replications.

In order to define the characteristics that need to be identical for a study to qualify as direct replication, the constraints on generality criteria (COG; Simons et al., 2017) are applied. The COG criteria provide a general scheme for which characteristics of study participants (the targeted population), study material and procedures, and the temporal specificity of an effect are necessary to be kept the same for a replication study to be an exact replication. Principles for choosing variables for the COG should be known empirical or theoretical boundary conditions, conditions that are tied to the substance of the study, and factors that experts consider to be important.

The taxonomy suggested by LeBel and colleagues (2018) combined with the COG results in a continuum from direct to conceptual replication that can be pre-specified. The dimensions underlying the classification of replication types should include procedural details (e.g., diagnostic instruments, blinding of assessors, unconcealed allocation/risk of bias), statistical methods, contextual variables (e.g., cultural context), therapist-related factors (manual adherence), and researcher-related factors (e.g., allegiance, conflicts of interest), all of which are also potential moderator variables.

Consider, for instance, a case in which a newly developed intervention for depression is first tested against a waitlist condition (WL) and is found to be superior. A subsequent study replicates the initial study, but compares the same intervention to treatment as usual (TAU). A direct replication of the newly developed intervention for depression would need to consist of a second comparison to WL, whereas the use of a different control condition (or treatment delivery in a natural setting, or applying the intervention over the internet etc.) constitutes a conceptual replication that already tells us something about the generalizability of the intervention effects and the relative efficacy of the new treatment. As another example, we might consider a case in which a new 12-session treatment for panic disorder is favorably tested against WL. A subsequent study also compares this new treatment to WL but uses a protocol that involves only 10 sessions, is conducted in a different country, and examines a slightly older patient population; and this second study does not find the treatment to be efficacious. Is this a failed replication study? Due to the lack of clear criteria, we are not currently able to provide a definitive answer to this question. With so many changes at once, we will never know why it did not replicate. Therefore, we need the changes to be decided on and documented more specifically; ideally, replication studies should change on one dimension at a time, so that differences in effects can be clearly attributed.

Incentives for authors for the use of a COG statement integrated into the taxonomy by LeBel and colleagues (2018) could be a protection from overly broad claims, a higher likelihood of successful replications, and inspiring follow-up studies that built upon the findings. Editors and reviewers could request a COG statement. Incentives for editors could be to have an equivalent measure to evaluate all papers, and for reviewers to have a measure for quality control, whereas for readers it helps to learn about the generality of the claims of a study (Simons et al., 2017).

#### 2. Study Characteristics That Increase the Trustworthiness of Results

Although some important methodological factors are included in current standards of study quality assessment, there is evidence that many intervention studies fall short of characteristics that increase the trustworthiness of results. Moreover, QRPs and publication bias distort the literature and limit the replicability of studies. In addition to the existing guidelines we propose to include the following issues:

- An assessment of reporting errors should be conducted. For consistency checks of *p* values, “statcheck” can be applied (Epskamp & Nuijten, 2016).
- Pre-registration should be mandatory. The study design and analysis plan need to be pre-specified and saved in a public registry or published prior to data collection. Pre-registration is a measure to enhance transparency, document timestamped decisions, helping to differentiate between confirmatory and exploratory analyses, and for reducing *p*-hacking and HARKing. Alternatively, registered reports (RRs) are a sensible publishing format that reduces QRPs and publication bias because in RRs the peer review is conducted prior to the data collection. This emphasizes the research question and the quality of methodology instead of the significance of the results (Chambers & Tzavella, 2022). Checklists for pre-registration and recommendations for RRs have been developed in the wider field of psychology to enhance the quality of reports and pre-registrations (Nuijten, Hartgerink, van Assen, Epskamp, & Wicherts, 2016). Developments in adjacent fields are ahead, such as in biomedical research where journals banded together to make registration mandatory (Siebert et al., 2020; ClinicalTrials.gov). Registered reports and replication reports are a promising format also for clinical psychological journals.
- A systematic assessment of whether the information provided in a pre-registration is sufficient should always be conducted and should be considered in the EST criteria or guidelines.
- It should be assessed whether the final study report matches the pre-registered plan. We do acknowledge, though, that this places an extra burden on reviewers, who need to spend more time reviewing a manuscript. To reduce this burden journals can invite specialized reviewers to specifically review open science aspects of the manuscript, such as whether the pre-registration matches the final study report or checking any shared materials.
- Open data and open materials should become standard to enhance transparency. Replication studies benefit to a large extent from open data and materials. However, it should be noted that open data and materials is not a prerequisite for replicating studies (Buzbas, Devezer, & Baumgaertner, 2023). If highly sensitive data present challenges to open data principles, restricted access to data, e.g. according to the different access categories of the German Psychological Association (DGPs^1^1https://zwpd.transmit.de/images/zwpd/dienstleistungen/ethikkommission/vorlage_opendata_v1.docx), is also a viable alternative. This is in line with the standards of the American Psychological Association (2020), which invites researchers to share their data. It should be motivated if data cannot be shared due to ethical or legal constraints, e.g. due to participant confidentiality or missing consent. Open material and sensitive material with restricted access can both be stored in repositories, such as the Open Science Framework (OSF; osf.io).

#### 3. Criteria for Successful Replication

As described in the taxonomy of replication, exact versus conceptual replication studies provide different information in case of replication success or failure. For example, when a conceptual replication study shows a failure of replication, this might be the result of hidden moderators. However, criteria are necessary for determining when (both direct and conceptual) replication studies are a success or failure. This conceptual issue has also not been explicitly addressed in mental health research to date, i.e. what defines a successful replication with respect to the statistical outcome of both the original and the replication study. That is, in addition to the definition of the study design as direct or conceptual replication, we propose criteria for the comparison of the quantitative results of an original and a replication study and the assessment of the replication of the study results as successful or failure, which are currently missing in research on psychological interventions.

Recent large-scale replication studies have proposed and comparatively evaluated different criteria, such as statistical significance, i.e., a study is deemed to be replicated if both the original study and the replication are statistically (non-)significant, or the direction of both effect estimates is the same (OSC, 2015). However, an application of criteria for (un)successful replication in research on psychological interventions is lacking (see also Nosek et al., 2022).

Given that multiple statistical options to determine replication success exist (OSC, 2015; Zwaan, Etz, Lucas, & Donnellan, 2018) and that there is no consensus for one particular method, we provide a short overview of the most relevant ones: Both original and replication studies are statistically (non-)significant, the direction of both effect estimates is the same, the original effect falls within the confidence interval of the replication, original and replication result are combined and significance is assessed (OSC, 2015), statistical consistency between the original study and replications is evaluated in multisite replication projects (Mathur & VanderWeele, 2020), the small telescopes approach (Simonsohn, 2015), sceptical *p*-value (Held, 2020), and replication Bayes factor (Ly, Etz, Marsman, & Wagenmakers, 2019). These criteria represent the currently most prominent options for evaluating replicability. Recently, a comparison of seven approaches (significance, small telescopes, classical and Bayesian meta-analysis, Bayes factor and replication Bayes factor, as well as skeptical *p*-value (Held, 2020) has been conducted (Muradchanian, Hoekstra, Kiers, & van Ravenzwaaij, 2021). According to the authors, Bayesian metrics as well as meta-analytic methods were found to perform slightly better than the other approaches in terms of true and false positives rates. That is, a positive replication result is observed when the underlying true effect is non-zero or when the true effect is practically zero under different levels of publication bias in a simulation study. When evaluating replicability in research on psychological interventions, we suggest applying multiple methods, all of which should be preregistered before conducting the study. Researchers should come to conclusions based on the results of all the methods, as they perform quite similarly. Moreover, applying more methods also provides more information.

All criteria presented in the three categories taxonomy of replication, study characteristics that increase the trustworthiness of results, and criteria for successful replication are provided in an info box (see Table 1). We exemplarily propose up to three specific criteria for each COG subdomain. This list is not exhaustive, because study designs and research foci differ considerably. We recommend that researchers adapt the COG specifically to the study designs that are utilized in their research domains.

**Table 1:** Info Box for Replication Studies in Clinical Psychology

Overall domains / Subdomains
1. Taxonomy of replication: Constraints on generality (COG)
Participants^a^ Diagnoses Symptom severity Comorbidity
Materials / stimuli^a^ Manual used Adherence to manual Therapist training / supervision
Procedure^a^ Primary and secondary outcomes Type of assessment (e.g., clinician-based vs. self-rated) Type of allocation
Historical / temporal specificity^b^ Changes in diagnostic criteria (e.g. in DSM) Common use of cellphones or internet access for app- and browser-based interventions / blended approaches
2. Study characteristics that increase the trustworthiness of results
Scales^c^ for quality assessment used (according to study type)
Are reporting errors absent in the study?
Preregistration Is a study pre-registered or is it a registered report? Are there sufficient details in the pre-registration/registered report? Do the analyses in the pre-registration match those in the final study report?
3. Criteria for successful replication: Methods to consider
Are the data and study materials openly available?
Are both original and replication study statistically significant?
Are the effect sizes of both the original and replication study in the same direction?
Does the effect size of the original study lie in the CI of the replication?
Is the meta-analytic effect size of combining the original and replication study statistically significant?
Is the effect size of the original study consistent with the replications in a multisite replication project (Mathur & VanderWeele, 2020)?
Small telescopes approach (Simonsohn, 2015): Is the replication effect size not significantly smaller than an effect size that would have 33% statistical power based on the sample size of the original study?
Replication Bayes factor (Verhagen & Wagenmakers, 2014; Wagenmakers, Verhagen, & Ly, 2016: Is there more evidence that the effect size of the replication is a null effect compared to the effect observed in the original study?

*Note.* DSM = Diagnostic and Statistical Manual; CI = Confidence interval.

^a^The proposed specific criteria are exemplary and not exhaustive. ^b^This category takes into account that norms and standards change over time, and studies should be evaluated according to the respective historical period. ^c^The quality assessment should be conducted according to the specific scale that is used.

### B. Evaluating Evidence on Efficacy of Interventions

Beyond establishing standards for future research, it is also important to understand which factors are causing the (non-)replicability of findings in the current literature by systematically investigating moderators of treatment effects. Specifically, the relative contributions of the different variables outlined in Section A to replication success (outcome) are of interest, e.g. study quality, the type of replication design, and contextual variables. Pre-registration and a taxonomy of replication should also be systematically integrated into the classification of ESTs, clinical guidelines, and meta-analyses to enhance the transparency and methodological comparability. In addition, differences between preregistered/replicated studies and other studies should be studied.

Moderator analyses can best be addressed with meta-analytic methods. For example, the efficacy of some interventions may be highly dependent on context variables, e.g., successful replication may only be demonstrated in very direct replication designs and may have low generalizability to different contexts. Other interventions may be more context-independent, with effects being replicated even in less strict settings regarding patient or therapist characteristics or modes of treatment delivery. That is, the criteria for replication outlined above should be related to the evaluation of studies as ESTs and considered when summarizing studies in meta-analyses. Importantly, findings from this line of research can then be useful to further refine the replication concept and criteria (A). For example, if a particular therapist characteristic is not relevant for determining the replicability, it no longer needs to be taken into account when evaluating whether a study is a direct or conceptual replication.

Moderators can also include variables that are typically used to address meta-scientific questions, for example whether a study was pre-registered or provides open data. Thus, investigating pre-registration as moderator in meta-analyses against the background of replicability can shed light on whether pre-registered studies differ from non-pre-registered studies not only in terms of treatment efficacy and study quality, but also in the replicability of their results.

### C. Planning and Conducting New Replication Studies

The new definitions and criteria (A) should be used to design future replication studies for psychological interventions in order to test the consistency of treatment effects by means of direct replication studies, as well as the generalizability of findings to varying contexts on the basis of an explicit taxonomy of replication. To guide future replication research, the taxonomy of different types of replication, including the relevant dimensions of similarity vs. dissimilarity of research design features and a COG statement, tailored to research on psychological interventions, should be applied. Researchers should start by directly replicating an original treatment effect in order to investigate whether the effect exists. Then, to examine the generalizability and detect hidden moderators, they should move on to conceptual replication studies, in which they modify important aspects of the study design (e.g., treatment manual used, characteristics of treatment delivery, definition of outcome, comparison condition, and contextual factors). Depending on how many and which variables in the COG are kept equal, the similarity of replication studies along the continuum from direct to conceptual replications should be varied. Thereby it can be determined in a direct replication whether an effect exists, and its boundary conditions and mechanisms can be identified in conceptual replications. Thus, the distinction between direct and conceptual replication studies will be helpful for assessing the heterogeneity of findings for a particular intervention. That is, conceptual replications will test whether the proposed constraints on generality are accurate, leading to a more refined understanding of the robustness of effects. A systematic program of research should evaluate how the size of an effect varies as a function of those constraints (Simons et al., 2018).

An important first step is to conduct an exact replication study to confirm the result of the original study. Second, in order to identify the most important hidden moderators assessed conceptual replications and also meta-analyses should be conducted, once a sufficient number of replication studies has been conducted where as rule-of-thumb can be used that 5 to 10 studies are needed per included moderator in a meta-analysis (van Houwelingen, Arends, & Stijnen, 2002). An agreed set of quality standards and criteria based on the COG concept that must be included in clinical trial reports should be established and constantly refined. The criteria and quality standards will inform future replication studies, and should also be taken into account by experts evaluating the current state of evidence of an intervention, e.g. when developing clinical guidelines or establishing EST.

In the long term, the adoption of COG statements will lead to a more cumulative understanding of the scope of the effects of psychological interventions.

## Conclusion

The current gold standard in evidence-based psychological treatments can be criticized for not paying sufficient attention to replicability. The current discussion surrounding replicability and reproducibility (Ioannidis, 2012; Munafò et al., 2017) offers the opportunity to define and potentially increase replicability also in mental health research. The development of an explicit concept and taxonomy of replication will enable the classification of studies investigating clinical interventions with respect to their similarity with original studies and will aid in planning and conducting replication studies in the future. The criteria themselves need to be continuously updated based on advances in replicability research in other areas and informed by emerging evidence regarding (moderators of) replicability in mental health research.

However, also a number of limitations have to be noted. Even if an effect is true, it is possible to fail to replicate due to seemingly innocuous differences in the implementation of the study (i.e. due to “hidden moderators”). Small variations in studies are unavoidable and exact replication is strictly impossible. Baribault and colleagues (2018) suggest to randomize variables that may be moderators of an effect in replication studies in order to test the robustness and generalizability of an effect. They propose a random selection of potential moderators, that is characteristics of the design that are not supposed to make a difference. If characteristics do not affect the results, this means that the results are more generalizable and to alter minor things should not matter. This is suggested for experimental research, e.g. different implementations of the same stimulus could be used to study whether the results are robust. However, as a large number of studies is necessary for this approach, it is not applicable to RCTs on psychological interventions. Compared to research in social psychology, studies in research on psychological interventions are much more costly and time-consuming, which makes it more difficult to study replicability. The question of how much money and effort researchers should spend on studying replicability given that conducting such studies is expensive in clinical psychology is related to the decision when to move on to other research topics, because studying replicability means at the same time that less scientific progress with respect to new findings will be made. This demonstrates that not all recommendations from social psychology are applicable in clinical psychology.

Based on this we would like to invite the readers to engage in discussions about the concrete criteria and next steps that we proposed. Designing replication studies should be based on empirical evidence and on theoretical predictions (Simons et al., 2018) and considered to be a collective research enterprise.
